# Transcriptome analysis of *Aedes albopictus* midguts infected by dengue virus identifies a gene network module highly associated with temperature

**DOI:** 10.1186/s13071-022-05282-y

**Published:** 2022-05-19

**Authors:** Zhuanzhuan Liu, Ye Xu, Yudi Li, Shihong Xu, Yiji Li, Ling Xiao, Xiaoguang Chen, Cheng He, Kuiyang Zheng

**Affiliations:** 1 Department of Pathogen Biology and Immunology, Jiangsu Key Laboratory of Immunity and Metabolism, Xuzhou Medical University, Xuzhou, China; 2 Department of Pathogen Biology, Key Laboratory of Tropical Disease Research of Guangdong Province, School of Public Health, Southern Medical University, Guangzhou, China; 3 Department of Pathogen Biology, Hainan Medical University, Haikou, Hainan China; 4 Taiyuan Central Hospital, Shanxi, China

**Keywords:** *Aedes albopictus*, Dengue virus serotype 2, Temperature, RNA sequencing, Gene correlation network analysis

## Abstract

**Background:**

Dengue is prevalent worldwide and is transmitted by *Aedes* mosquitoes. Temperature is a strong driver of dengue transmission. However, little is known about the underlying mechanisms.

**Methods:**

*Aedes albopictus* mosquitoes exposed or not exposed to dengue virus serotype 2 (DENV-2) were reared at 23 °C, 28 °C and 32 °C, and midguts and residual tissues were evaluated at 7 days after infection. RNA sequencing of midgut pools from the control group, midgut breakthrough group and midgut nonbreakthrough group at different temperatures was performed. The transcriptomic profiles were analyzed using the R package, followed by weighted gene correlation network analysis (WGCNA) and Kyoto Encyclopedia of Genes and Genomes (KEGG) analysis to identify the important molecular mechanisms regulated by temperature.

**Results:**

The midgut infection rate and midgut breakthrough rate at 28 °C and 32 °C were significantly higher than those at 23 °C, which indicates that high temperature facilitates DENV-2 breakthrough in the *Ae. albopictus* midgut. Transcriptome sequencing was performed to investigate the antiviral mechanism in the midgut. The midgut gene expression datasets clustered with respect to temperature, blood-feeding and midgut breakthrough. Over 1500 differentially expressed genes were identified by pairwise comparisons of midguts at different temperatures. To assess key molecules regulated by temperature, we used WGCNA, which identified 28 modules of coexpressed genes; the ME3 module correlated with temperature. KEGG analysis indicated that RNA degradation, Toll and immunodeficiency factor signaling and other pathways are regulated by temperature.

**Conclusions:**

Temperature affects the infection and breakthrough of *Ae. albopictus* midguts invaded by DENV-2, and *Ae. albopictus* midgut transcriptomes change with temperature. The candidate genes and key pathways regulated by temperature provide targets for the prevention and control of dengue.

**Graphical abstract:**

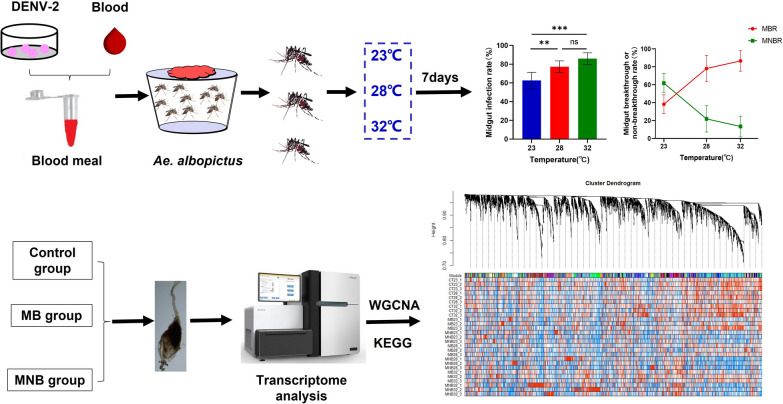

**Supplementary Information:**

The online version contains supplementary material available at 10.1186/s13071-022-05282-y.

## Background

Dengue fever is an arthropod-borne disease caused by the dengue virus (DENV) [1]. In general, clinical manifestations vary after infection with DENV. Most people are asymptomatic or have only minor symptoms, including fever, rash, and body pain. However, a proportion of patients develop severe dengue, which manifests as severe bleeding, shock, and even death [2]. Although dengue is endemic worldwide, it is distributed mainly in tropical and subtropical regions. It is estimated that approximately 400 million people are infected with DENV globally every year, with 25% of this population presenting clinical symptoms [3]. In recent years, dengue outbreaks have appeared in the Americas, Bangladesh, the Philippines, and Nepal [4–7]. In China, dengue frequently occurs in the provinces of Guangdong, Guangxi, and Yunnan. In 2014, the largest outbreak was reported in Guangdong Province, with 47,056 cases and six deaths [8, 9]. The area endemic for dengue is constantly expanding, and the number of people at risk increases yearly.

Dengue is transmitted by mosquitoes of the genus *Aedes*, such as *Aedes aegypti* and *Aedes albopictus*. *Aedes aegypti* is internationally recognized as the principal vector of dengue; *Ae. albopictus* is the secondary vector [10]. In China, the distribution of *Ae. aegypti* is limited to only a few areas of Guangdong, Hainan and Yunnan, and *Ae. albopictus* is the dominant mosquito species that causes dengue outbreaks in China; *Ae. albopictus* is distributed in perennially warm provinces and the north, southwest and southeast coastal areas [11]. Moreover, the geographical distribution of *Ae. albopictus* is continuing to expand with the acceleration of global warming, urbanization and trade, with an increased risk of the spread of dengue.

Temperature affects the prevalence of dengue [12, 13], and in the absence of measures to mitigate climate change, an additional 7.5 million dengue cases per year could occur. If the increase in temperature remains within 2 °C, the annual increase in dengue cases could be reduced by 1–3 million; if it is controlled to within 1.5 °C, the number of cases could be further reduced [14]. In general, the risk of dengue increases with increasing temperature, which is primarily associated with improving the vector competence of mosquitoes to transmit DENV. In one study, when the temperature was higher than 26 °C, the extrinsic incubation period of DENV in *Ae. aegypti* was approximately 1 week, but was longer when the temperature was lower than 21 °C; when the temperature was lower than 18 °C, *Ae. aegypti* could not transmit DENV [15]. Similar results were shown for *Ae. albopictus* [16]. Nevertheless, the mechanism by which temperature affects the transmission of DENV by *Aedes* remains unclear.

Mosquito-borne viruses must overcome a mosquito’s midgut and salivary gland barrier to replicate; indeed, the midgut is the first barrier against a virus. Thus, the equilibrium of the midgut environment affects viral replication [17, 18]. For example, in *Ae. aegypti* infected with Zika virus and cultured at 20 °C, 28 °C, and 36 °C, the gene expression profiles of the midgut change with temperature, especially under low-temperature conditions (20 °C), with significant alterations in gene expression related to blood digestion, active oxygen metabolism and innate immunity [19].

Our previous research showed that DENV serotype 2 (DENV-2) is confined to the midgut of *Ae. albopictus* and slowly proliferates at 18 °C; when the temperature is 23–32 °C, DENV-2 breaks through the midgut barrier of *Ae. albopictus* and invades the salivary glands [16]. To better understand the interaction between *Ae. albopictus* and DENV-2 under different temperatures, the infection status in the midgut and residual tissues of *Ae. albopictus* at 23 °C, 28 °C and 32 °C was investigated. Transcriptome sequences were obtained from the midgut of *Ae. albopictus*. In addition, gene network modules highly related to temperature were identified using weighted gene correlation network analysis (WGCNA).

## Methods

### Mosquitoes

*Aedes albopictus* were collected in Foshan city, Guangdong Province, China, and bred in a standardized room (constant 27 ± 1 °C, 70–80% relative humidity and a 16 h:8 h light–dark photoperiod). The eggs hatched into larvae in dechlorinated water, and the larvae were fed turtle food until the pupal stage. The pupae were transferred into a cup and placed in a mosquito cage. Adults emerged during a period of 2–3 days and were fed 10% glucose. The mosquitoes were fed defibrinated sheep blood for egg production.

### The proliferation of DENV

Experiments with DENV-2 were performed in a biological safety cabinet. DENV-2 (New Guinea C) proliferated in C6/36 cells. C6/36 cells cultured in RPMI-1640 medium containing 10% fetal bovine serum were inoculated with DENV-2 at a multiplicity of infection of 1; the culture flask was gently shaken for 15 min at room temperature and subsequently incubated at 37 °C and 5% CO_2_ for 2 days. The supernatant was harvested after centrifugation at 1500× *g* for 5 min. The DENV-2 titer was determined based on the 50% tissue culture infective dose (TCID_50_) [20].

### Mosquito infection with DENV-2

The mosquito infection experiment was performed in a biosafety level 2 laboratory. Five- to 7-day-old female *Ae. albopictus* mosquitoes were starved for 16–20 h. Fresh DENV-2 (8.625 log_10_ TCID_50_/mL) was mixed with defibrinated sheep blood at a ratio of 2:1. After incubation at 37 °C for 30 min, the blood meal was transferred into a Hemotek blood reservoir unit (Discovery Workshops, Lancashire, UK). *Aedes albopictus* mosquitoes were allowed to feed on the blood meal for 30 min. The engorged mosquitoes were anesthetized with CO_2_, placed in 250-mL paper cups covered with gauze (10 mosquitoes/cup), and maintained in different incubators precisely set at 23 °C, 28 °C and 32 °C, 80% relative humidity and a 16 h:8 h (light:dark) photoperiod. For the control (CT) group, DENV-2 was replaced with RPMI-1640/2% fetal bovine serum. All mosquitoes were fed 10% glucose solution. Three batches of mosquito infections were performed for each temperature.

### DENV-2 detection

The midgut and residual tissues of each mosquito were dissected and individually examined at 7 days post-infection. The tissues from 466 mosquitoes that had ingested viral blood meals and 90 mosquitoes comprising the CT groups were tested for DENV-2 infection. Total RNA was extracted according to the manufacturer’s protocol (Promega, Madison, WI). Complementary DNA (cDNA) was synthesized using 4 µL of RNA and random primers following the manufacturer’s recommendations for the GoScript Reverse Transcription System (Promega). DENV-2 positivity was determined by polymerase chain reaction (PCR) using previously described primers (forward primer, 5ʹ-TCAATATGCTGAAACGCGCGAGAAACCG-3ʹ; reverse primer, 5ʹ-TTGCACCAACAGTCAATGTCTTCAGGTTC-3ʹ) [21]. PCR was performed according to the manufacturer’s protocol for the Maxima Hot Start Green PCR Master Mix (Thermo Fisher Scientific, Waltham, MA). The 511-bp target fragment was identified by 1% agarose gel electrophoresis. The residual RNA was stored at − 80 °C.

### Determination of the midgut infection, midgut breakthrough and midgut nonbreakthrough rates of *Ae. albopictus*

When DENV-2 was simultaneously detected in the midgut and the residual tissue of *Ae. albopictus*, it was considered to have broken through the midgut barrier of the mosquitoes; these mosquitoes constituted the midgut breakthrough (MB) group. When DENV-2 was detected only in the midgut and not in residual tissue, it was considered to have not broken through the midgut barrier; these mosquitoes constituted the midgut nonbreakthrough (MNB) group. The midgut infection rate (MIR), midgut breakthrough rate (MBR), and midgut nonbreakthrough rate (MNBR) of *Ae. albopictus* were determined using the following formulas:

$$ {\text{MIR}} =\, {\text{number of positive midguts}}/{\text{total number of midguts tested}} \times {1}00\% $$

$$ {\text{MBR}} = {\text{number of mosquitoes with a positive midgut and residual tissue}}/{\text{number of positive midguts }} \times {1}00\% $$

$$ {\text{MNBR}} = {\text{number of mosquitoes with a positive midgut but negative residual tissue}}/{\text{number of positive midguts}} \times {1}00\% $$

### Library preparation and RNA sequencing

Midguts from the control (CT), MB and MNB groups at each temperature (23 °C, 28 °C and 32 °C) were selected and divided into nine groups according to the results of the above experiment. The quality and integrity of a single midgut were tested using a 2100 Bioanalyzer (Agilent, Santa Clara, CA); midgut RNA concentrations were detected using a Nanodrop 2000. Three midgut samples of each group were mixed to form a pool, and each group had three biologically repeated pools. The biological replicates of midgut pools were from different feeding batches. In total, 27 pools were sent to Wuhan Huada Medical Laboratory for RNA sequencing. Total RNA was purified with the messenger RNA enrichment method or ribosomal RNA removal method, and a cDNA library was constructed. The prepared libraries were sequenced with DNASeqPE150 (BGI, China).

### RNA sequence analysis in response to temperature and DENV-2 exposure

The raw data obtained by RNA sequencing were filtered to remove low-quality data, contaminating linkers, and a high N content of unknown bases. The filtered data, as clean reads, were compared with the genome of the Foshan strain of *Ae. albopictus* (AaloF1, VectorBase, https://www.vectorbase.org), and novel transcripts were analyzed. Principal component analysis (PCA) was used to evaluate the sample repeatability and overall differences between samples. The gene length and total reads were normalized. Relative expression levels were assessed as fragments per kilobase of transcript per million fragments mapped (FPKM). Intragroup and intergroup Pearson correlation coefficients were calculated according to FPKM values, and a heat map was drawn.

### Identification of differentially expressed genes and coexpression network modules

The differentially expressed genes (DEGs) were analyzed and visualized using the Ballgown package (version 2.16.0) and ggplot2 (version 3.3.5) in R (version 4.0.5) [22]. The DEGs of the MB and MNB groups at each temperature and those between two adjacent temperatures in the MB or MNB groups are illustrated by a Venn diagram. DEGs were identified as genes with twofold or more changes between samples and a false discovery rate (FDR) < 0.05. WGCNA of all genes (FPKM > 1) was performed using the WGCNA (version 1.69) package [23]. An adjacency matrix of the genes’ similarity was conducted by pairwise Pearson correlation analysis. Using an appropriate soft threshold, *β* = 4 was obtained based on the pickSoftThreshold function. The cluster dendrogram plot and clustering tree of coexpression gene modules of all genes were constructed with the parameters of cutHeight = 0.75 and minSize = 30. Module-trait relations were evaluated through module eigengenes (MEs). The relationships between MEs and traits (temperature, treatment with or without virus, and tested positive or negative) were visualized through a heat map, and assessed by *P*-values and linear regression between gene expression profile and each trait. Modules significantly associated with the traits were identified by |cor | > 0.5 and *P* < 0.05 and considered to be the key modules. Gene ontology (GO) and Kyoto Encyclopedia of Genes and Genomes (KEGG) pathway enrichment analyses of the genes in key modules were analyzed using ClusterProfiler version 4.1 [24].

### Quantitative real-time PCR

The genes regulated by temperature were validated by quantitative real-time PCR (qRT-PCR). The levels of 18 genes were determined in the midguts at different temperatures. RNA and cDNA were synthesized using the above-described protocol. qRT-PCR was performed in triplicate for each sample with SYBR Green Mix (Yeasen, Shanghai, China). Gene expression was normalized to that of * RPS7*. The primer sequences are listed in Additional file 3: Table S1. The program was 95 °C for 5 min, followed by 40 cycles of 95 °C for 10 s, 60 °C for 10 s and 72 °C for 10 s.

### Data analysis

All statistical analyses were performed with SPSS 20.0 (IBM, Chicago, IL). MIR, MBR, and MNBR for *Ae. albopictus* infected with DENV-2 were separately compared at different temperatures using chi-square (and Fisher’s exact) tests. *P*-values were corrected by Bonferroni adjustment. The interaction between temperature and viral ability to escape midgut was determined by a multivariate ANOVA. The expression levels of genes were analyzed using the Kruskal–Wallis *H*-test. *P* < 0.05 was considered statistically significant.

## Results

### Temperature affects DENV-2 infection and midgut breakthrough in *Ae. albopictus*

**Fig. 1a, b:**
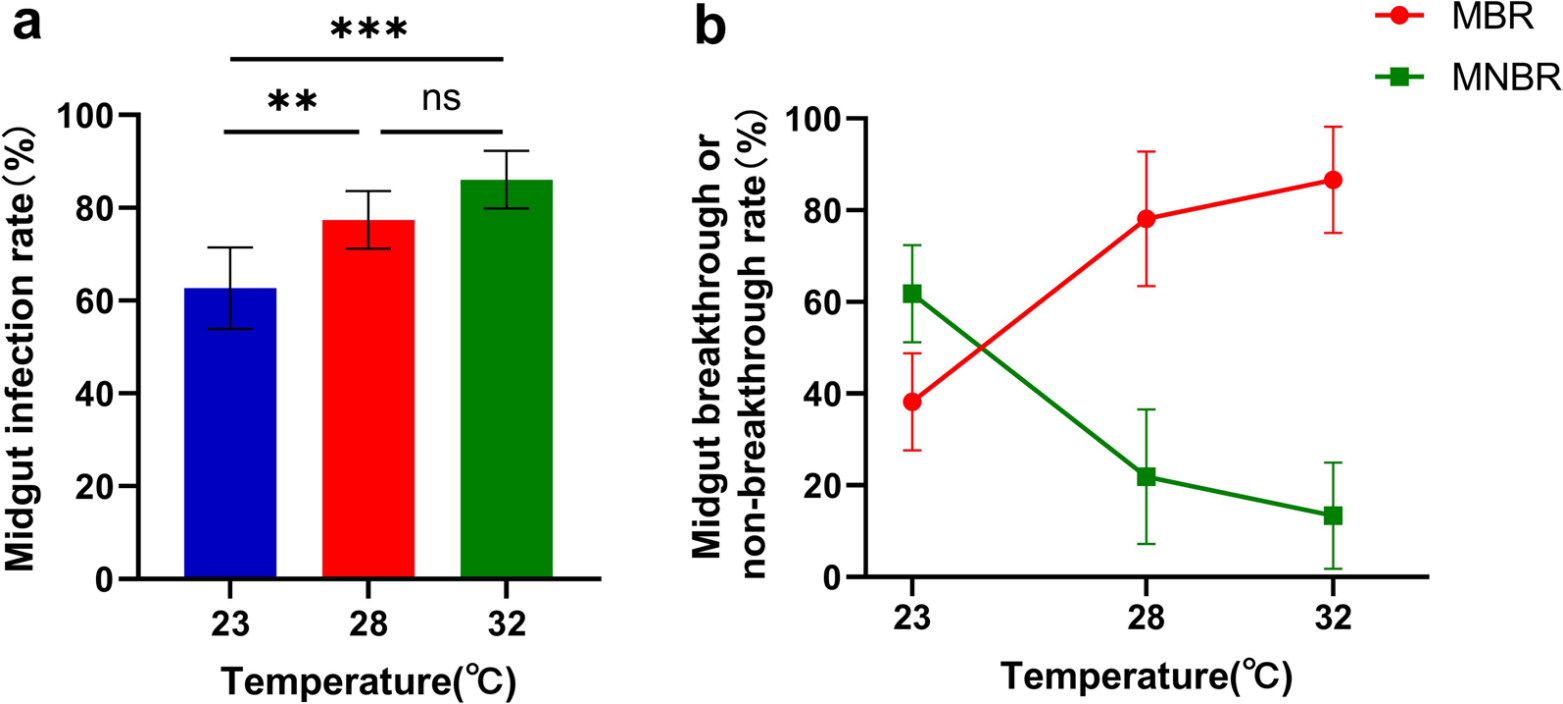
Analysis of the midgut of *Aedes albopictus* infected with dengue virus serotype 2 (DENV-2) at different temperatures. *Aedes albopictus* orally infected with DENV-2 were reared at 23 °C, 28 °C and 32 °C. The midguts and residual tissue were dissected at 7 days post-infection, and DENV-2 was detected by polymerase chain reaction (PCR). **a** Midgut infection rate. **b** Analysis of midgut breakthrough [midgut breakthrough rate (*MBR*), midgut nonbreakthrough rate (*MNBR*)] in *Ae. albopictus* at different temperatures*.* The error bars represent the 95% confidence interval. ** *P* < 0.01, *** *P* < 0.001,* ns* nonsignificant

The DENV-2 titer was determined to be 8.625 log_10_ TCID_50_/mL. In total, 466 *Ae. albopictus* mosquitoes that ingested blood meals containing DENV-2 were used to analyze the midgut infection and breakthrough rates at 23 °C, 28 °C and 32 °C. The MIR was significantly higher at 28 °C and 32 °C than at 23 °C (*χ*^2^ = 9.658, *P* < 0.01; *χ*^2^ = 16.909, *P* < 0.001) (Fig. 1a). Although the MIR was higher at 32 °C than at 28 °C, there was no significant difference between the two groups (*χ*^2^ = 3.001,* P* = 0.107) (Fig. 1a). The MBR of *Ae. albopictus* infection by DENV-2 was closely related to temperature and gradually increased when the temperature increased, although the MNBR gradually decreased. The MBR was significantly higher at 28 °C and 32 °C than at 23 °C (*P* < 0.01, *P* < 0.001, respectively), whereas the MNBR had the opposite trend (Fig. 1b). The MNBR was significantly higher than the MBR at 23 °C, but the MBR was significantly higher than the MNBR at 28 °C and 32 °C (Fig. 1b). The interaction between temperature and viral ability to escape the midgut was determined by a multivariate ANOVA. The result showed that temperature had a significant effect on viral ability to escape midgut.

### Characterization of *Ae. albopictus* transcriptomes at different temperatures

Transcriptome sequencing was performed on 27 samples from the CT, MB, and MNB groups at different temperatures (23 °C, 28 °C and 32 °C). Clean reads were obtained after removing low-quality data, and trimmed reads were obtained. Nearly 80% of the reads were mapped to the *Ae. albopictus* Foshan strain genome (Table 1).

**Table 1:** Overview of the mapping of RNA sequencing reads

Sample name	Accession number	Total reads	Trimmed paired reads	Trimmed reads mapped to the genome	Mapping rate (%)
CT23-1	SRR16503765	54,772,829	43,027,438	44,830,001	81.85
CT23-2	SRR16503764	53,395,633	42,115,248	43,759,069	81.95
CT23-3	SRR16503753	54,865,514	43,008,208	45,189,103	82.36
CT28-1	SRR16503745	55,807,603	42,799,344	45,686,153	81.86
CT28-2	SRR16503744	54,595,956	42,754,238	44,446,199	81.41
CT28-3	SRR16503743	55,657,054	43,167,914	45,847,667	82.38
CT32-1	SRR16503742	54,596,339	43,143,450	44,902,572	82.24
CT32-2	SRR16503741	54,575,842	43,233,636	44,911,335	82.29
CT32-3	SRR16503740	53,828,160	43,116,042	44,519,031	82.71
MB23-1	SRR16503739	55,680,172	43,272,084	45,751,800	82.17
MB23-2	SRR16503763	55,593,162	43,166,298	46,254,004	83.20
MB23-3	SRR16503762	53,854,014	43,234,376	43,751,782	81.24
MB28-1	SRR16503761	54,532,721	42,916,424	44,240,737	81.13
MB28-2	SRR16503760	55,082,520	43,313,808	45,303,233	82.25
MB28-3	SRR16503759	53,442,771	42,876,264	43,282,494	80.99
MB32-1	SRR16503758	54,496,832	43,216,764	44,790,010	82.19
MB32-2	SRR16503757	54,009,938	43,101,818	44,081,104	81.62
MB32-3	SRR16503756	53,385,010	42,789,656	43,476,908	81.44
MNB23-1	SRR16503755	55,517,642	42,595,030	46,663,792	84.05
MNB23-2	SRR16503754	54,594,901	42,346,994	44,982,402	82.39
MNB23-3	SRR16503752	55,500,160	42,492,630	46,458,788	83.71
MNB28-1	SRR16503751	56,410,592	43,144,664	47,226,360	83.72
MNB28-2	SRR16503750	54,893,018	43,252,930	45,876,636	83.57
MNB28-3	SRR16503749	54,651,677	42,863,124	45,496,019	83.25
MNB32-1	SRR16503748	55,653,684	43,066,758	45,287,828	81.37
MNB32-2	SRR16503747	53,792,991	42,047,606	45,040,839	83.73
MNB32-3	SRR16503746	53,760,318	43,236,118	43,159,101	80.28

* CT* Control,* MB* midgut breakthrough, *MNB* midgut nonbreakthrough

**Fig. 2a–d:**
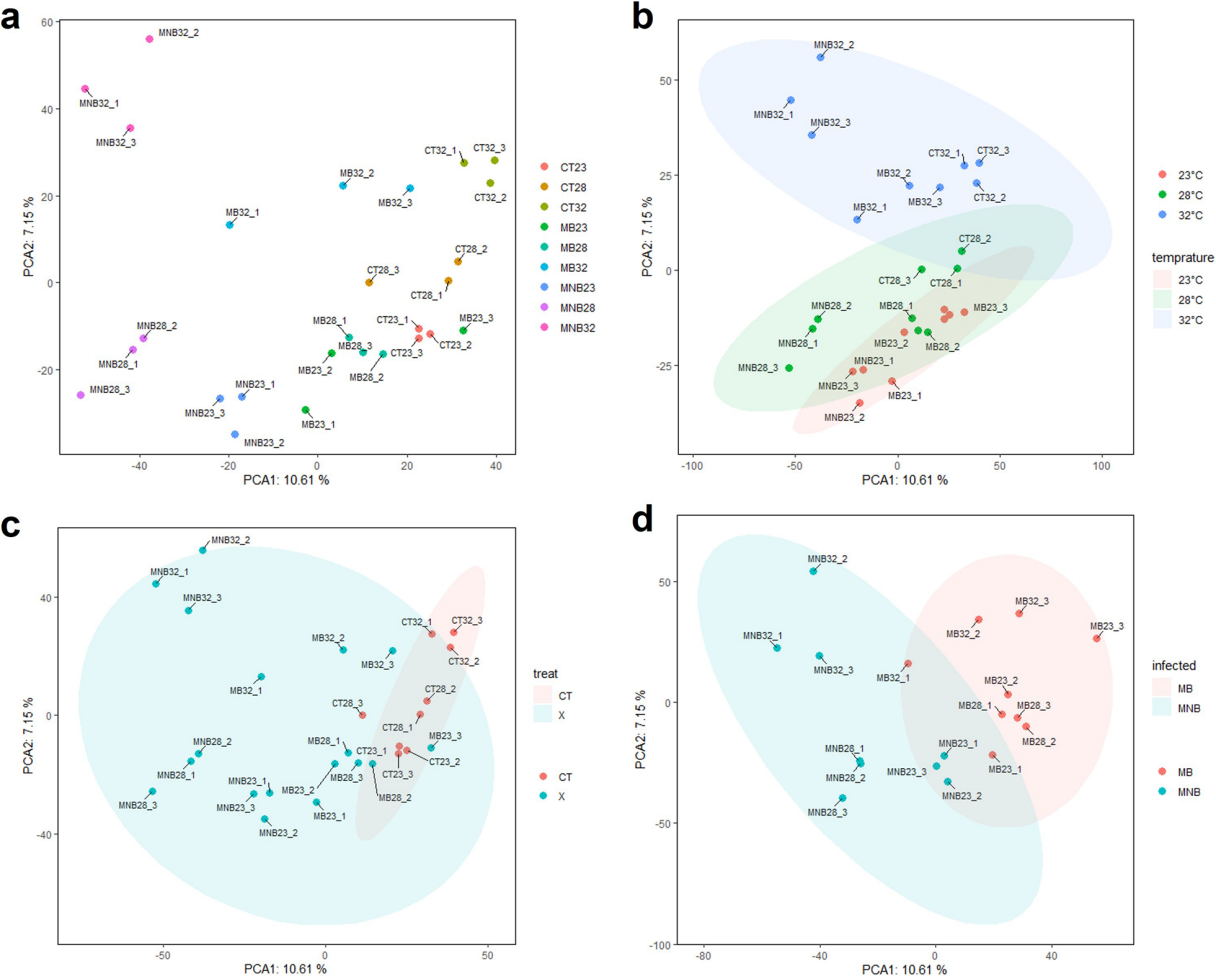
Principal component analysis (PCA) of the general transcriptome characteristics of *Aedes albopictus.* PCA1 and PCA2 accounted for 10.61% and 7.15% of the total variance in the dataset, respectively. Cluster analysis of transcriptome data from a single sample (**a**), samples at different temperatures (**b**), midgut datasets associated with viral treatment (**c**), and midgut breakthrough status (**d**).* CT* Control,* MB* midgut breakthrough, *MNB* midgut nonbreakthrough

PCA was used to analyze the variance of within-group samples and gene expression patterns associated with temperature, infection and breakthrough of the midgut. The PCA plot showed a high degree of reproducibility among replicate samples (Fig. 2a), and gene expression datasets of midguts clustered at different temperatures (Fig. 2b). Overall, the expression profiles of the midgut transcripts changed in response to blood-feeding between the CT group and the infection groups (Fig. 2c), and the clustering of transcript profiles was affected by midgut breakthrough (Fig. 2d). Furthermore, we analyzed Pearson correlation coefficients among all the samples, and a heat map showed correlation values of paired samples between 0.75 and 1 (Additional file 1: Fig. S1).

### Temperature alters *Ae. albopictus* transcriptomes during DENV-2 infection

**Fig. 3a–f:**
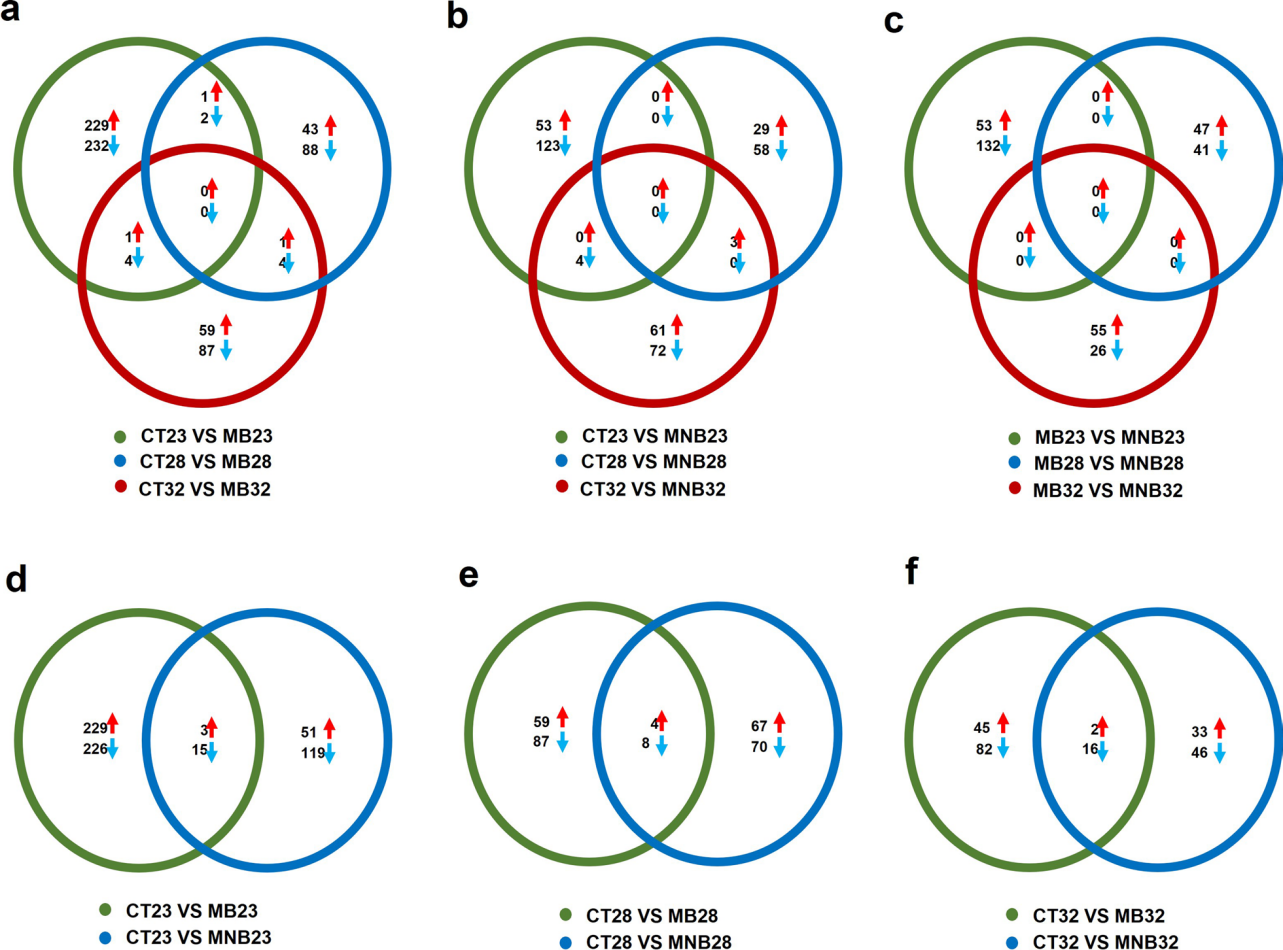
Venn diagram representation of the number of differentially expressed genes (DEGs) identified by RNA sequencing analysis in the midguts of *Aedes albopictus.* Number of DEGs between the CT and MB groups (**a**), CT and MNB groups (**b**), and MB and MNB groups (**c**) at different temperatures; **d** the MB and MNB groups compared to the CT group at 23 °C; **e** the MB and MNB groups compared to the CT group at 28 °C; **f** the MB and MNB groups compared to the CT group at 32 °C. For other abbreviations, see Fig. 2

DEGs between the CT and MB groups were analyzed at each temperature. There were 469 DEGs at 23 °C, 139 at 28 °C and 156 at 32 °C, and the same three upregulated and 10 downregulated genes at two of the temperatures (Fig. 3a). Compared to DEGs between the CT and MB groups, the transcriptomes between the CT and MNB groups were altered, with 180 DEGs at 23 °C, 90 at 28 °C and 140 at 32 °C, and the same three upregulated genes and four downregulated genes at two of the temperatures (Fig. 3b). Since temperature is an important factor that affects the midgut barrier, we analyzed the MB and MNB DEGs at different temperatures. Most DEGs were observed at 23 °C, followed by 28 °C, with the lowest number at 32 °C. There were no overlapping genes among the groups (Fig. 3c). The data show that the *Ae. albopictus* midgut transcriptomes change in response to temperature.

In addition, the DEGs were different between the MB and MNB groups compared to the CT group at the same temperature. At 23 °C, there was a notable difference with 231 upregulated and 241 downregulated genes between the CT and MB groups, far more than between the CT and MNB groups (Fig. 3d). At 28 °C, the numbers of DEGs between the CT and MB groups were almost identical to those between the CT and MNB groups (Fig. 3e). At 32 °C, there were 45 upregulated and 82 downregulated genes between the CT and MB groups, and 33 upregulated and 46 downregulated genes between the CT and MNB groups (Fig. 3f). These results demonstrate that the *Ae. albopictus* midgut transcriptome is affected by the midgut barrier.

### Key gene network associated with DENV-2 infection identified by WGCNA

**Fig. 4a–d:**
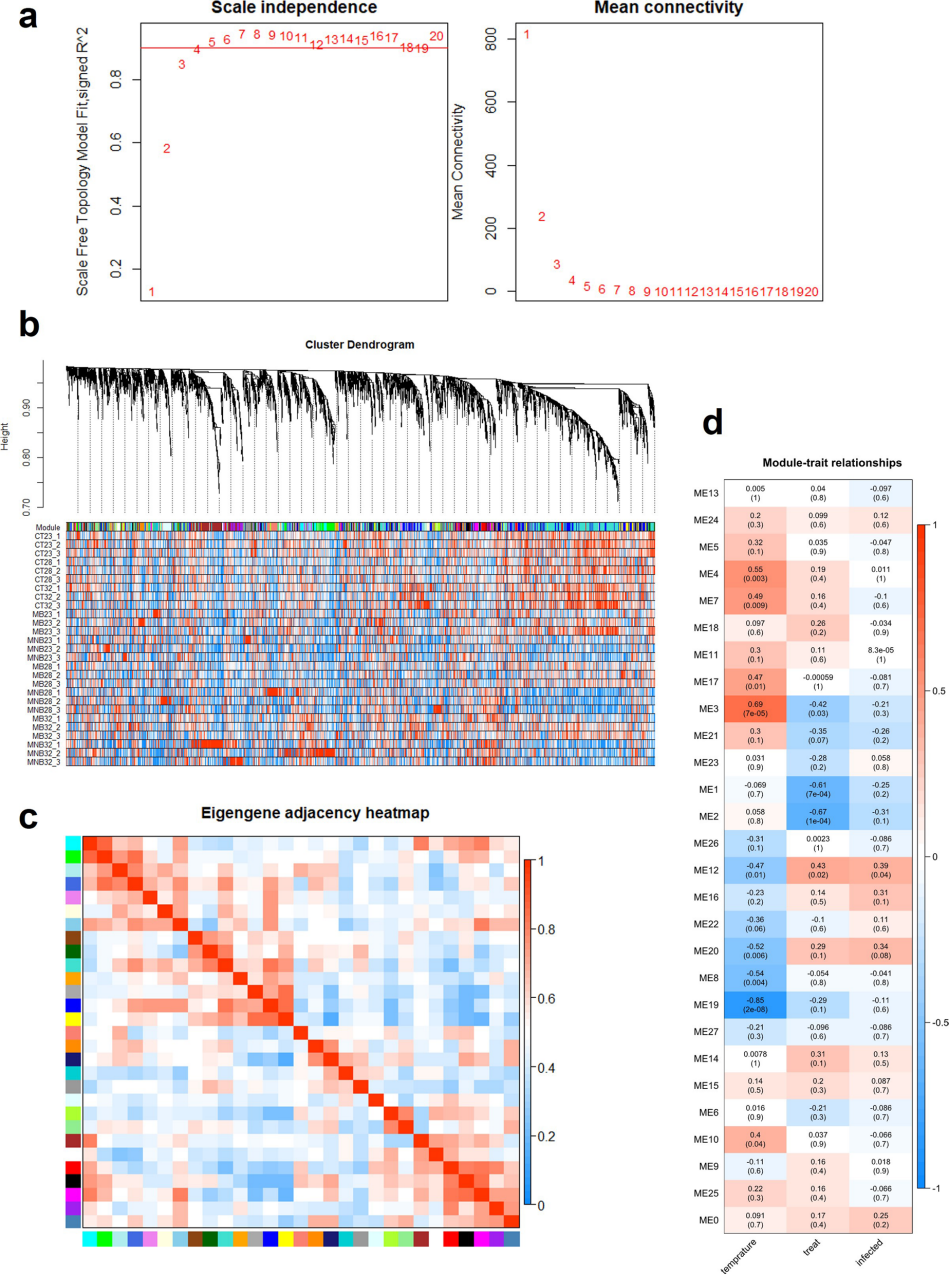
Weighted gene correlation network analysis revealing gene coexpression networks. **a** Analysis of network topology for various soft-thresholding powers. **b** The hierarchical cluster tree shows 28 modules of coexpressed genes. **c** The eigengene adjacency heat map indicates the relationship between the pairwise gene modules. **d** Correlations between modules and temperature. Each unit comprises the weight correlation coefficients and *P*-values. *ME* Module eigengene; for other abbreviations, see Fig. 2

To construct a WGCNA network, we first calculated the thresholding power to perform network topology analysis. The thresholding power was set at 4 in WGCNA because the scale independence reached 0.9 and showed suitable mean connectivity (Fig. 4a). Modules were determined using the WGCNA R package. The hierarchical cluster tree showed 28 modules of coexpressed genes (Fig. 4b), and the eigengene adjacency heat map indicated a relationship between pairwise gene modules (Fig. 4c). Correlations between modules and temperature and ingestion of DENV-2 blood meal were analyzed by WGCNA. According to the results, the ME3 module had the highest correlation with temperature, and the ME12 module was highly associated with the ingestion of a viral blood meal (Fig. 4d).

### Functional analysis of the key module associated with temperature

**Fig. 5a, b:**
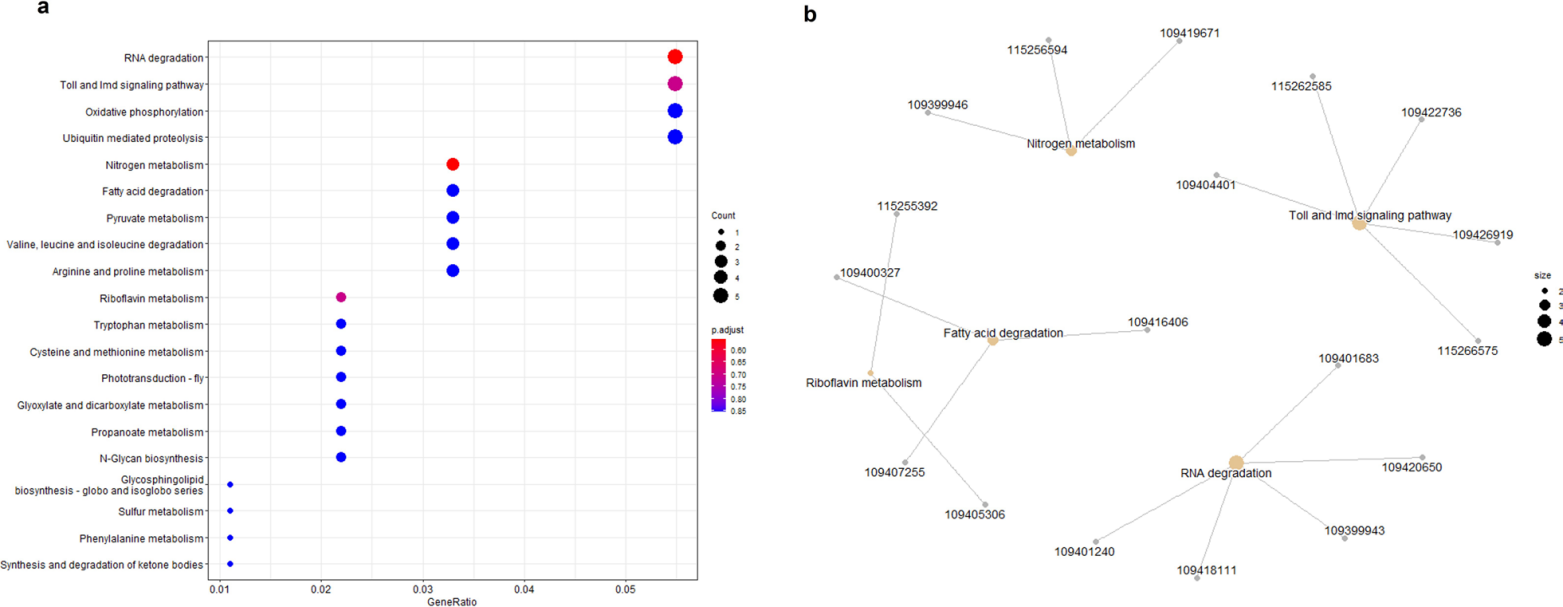
Functional analysis of the key module associated with temperature. **a** Kyoto Encyclopedia of Genes and Genomes enrichment analysis of DEGs from the ME3 module. **b** Genes corresponding to the partial pathway regulated by temperature

In this study, we focused on the pathway by which temperature affected the transmission of DENV-2 by *Ae. albopictus*. The ME3 module correlated with temperature was evaluated by GO and KEGG analyses. Unfortunately, no significant GO terms were enriched in this module (Additional file 2: Fig. S2). In the KEGG analysis, RNA degradation, Toll and immunodeficiency factor (IMD) signaling pathways and other pathways were enriched. Hence, our results show that these pathways are regulated by temperature (Fig. 5a). The genes involved in RNA degradation, Toll and IMD signaling pathways, nitrogen metabolism, fatty acid degradation and riboflavin metabolism are listed in Table 2 (Fig. 5b).

**Table 2:** Module genes regulated by temperature

Module-regulated pathway	Gene number	Protein
RNA degradation	109401683	m7GpppN-messenger RNA hydrolase
	109420650	U6 small nuclear RNA-associated Sm-like protein LSm4
	109399943	CCR4-NOT transcription complex subunit 6-like
	109418111	Protein PAT1 homolog 1
	109401240	Heat shock 70-kDa protein cognate 5
Toll and IMD	109404401	Modular serine protease-like
	115262585	Uncharacterized LOC115262585
	109422736	Peptidoglycan-recognition protein LB-like
	109426919	Uncharacterized LOC109426919
	115266575	Myeloid differentiation primary response protein 88-like
Nitrogen metabolism	109399946	Glutamine synthetase 2 cytoplasmic
	115256594	Carbonic anhydrase 1-like
	109419671	Carbonic anhydrase 1-like
Fatty acid degradation	109400327	Very long-chain specific acyl-CoA dehydrogenase, mitochondrial-like
	109416406	Acetyl-CoA acetyltransferase, cytosolic-like
	109407255	Carnitine *O*-palmitoyltransferase 2, mitochondrial-like
Riboflavin metabolism	115255392	Testicular acid phosphatase
	109405306	Flavin reductase (NADPH)

*IMD* Immunodeficiency factor

### Validation of hub genes regulated by temperature

**Fig. 6:**
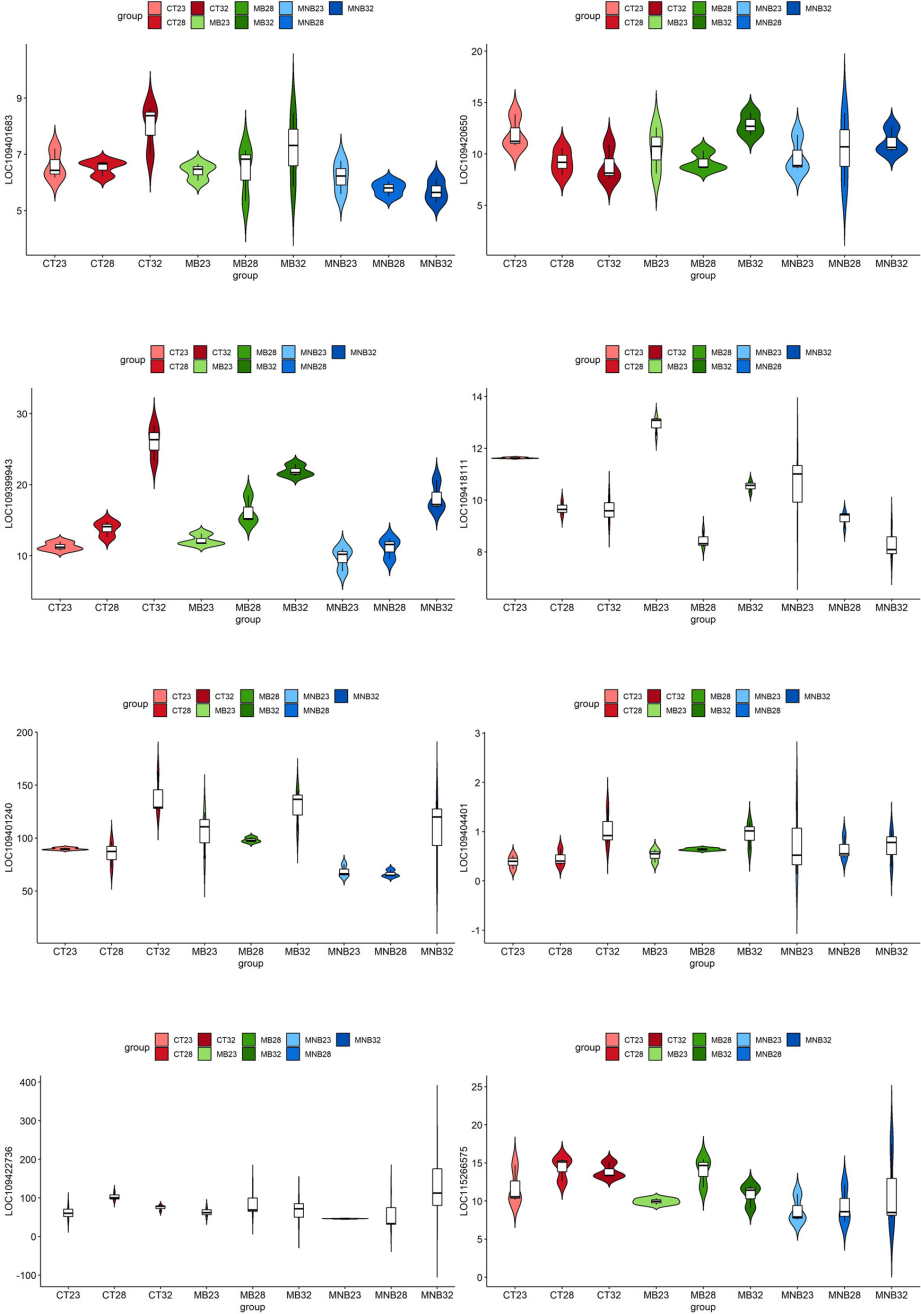
RNA sequencing analysis of the expression of hub genes regulated by temperature. For abbreviations, see Figs. 2 and 3

**Fig. 7:**
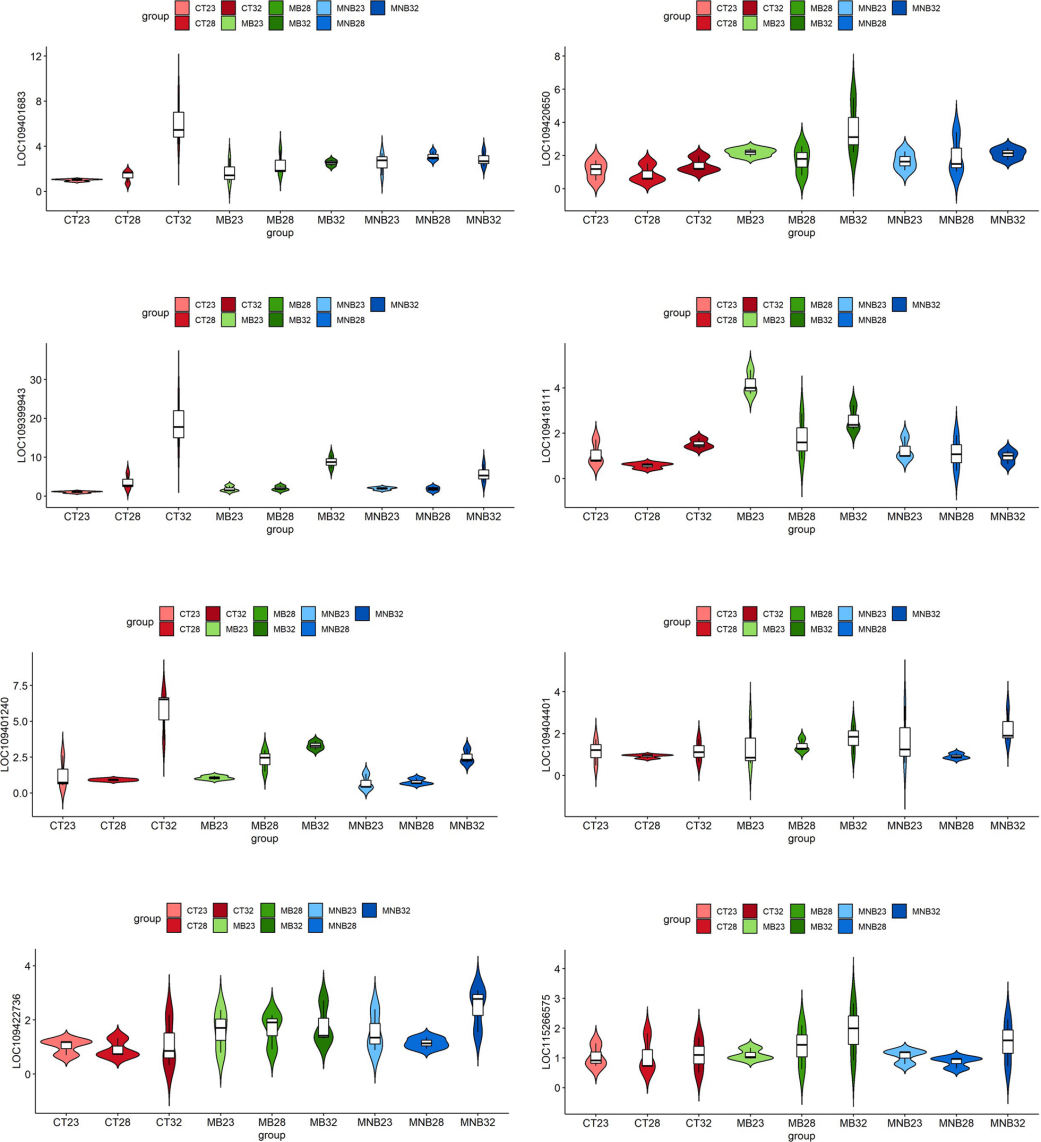
Quantitative real-time PCR validation of the expression of hub genes regulated by temperature. For abbreviations, see Figs. 2 and 3

We selected eight genes related to the antiviral immunity of mosquitoes, as indicated by the literature, for validation. The expression levels of these genes were analyzed from RNA sequencing datasets (Fig. 6) and determined by qRT-PCR (Fig. 7). The expression trends of most samples were similar according to the RNA sequencing and qRT-PCR. The Kruskal–Wallis *H*-test demonstrated that “LOC109399943” annotated as “CCR4-NOT transcription complex subunit 6-like”, “LOC109418111” annotated as “protein PAT1 homolog 1” and “LOC109401240” annotated as “heat shock 70-kDa protein cognate 5” were significantly different in the samples overall. These hub genes regulated by temperature represent targets for dengue prevention.

## Discussion

Model predictions and laboratory studies have shown that temperature affects the vector competence of *Ae. albopictus* for the transmission of DENV [16, 25]. However, little is known about the underlying mechanisms. In this study, RNA sequencing of the midguts of *Ae. albopictus* in the CT, MB and MNB groups was conducted, and the results revealed different transcriptional variations in response to DENV infection and temperature. WGCNA was used to identify key gene networks regulated by temperature. Then, we determined hub pathways associated with temperature.

In this research, we collected *Ae. albopictus* mosquitoes with different infection statuses. For the simultaneous collection of mosquitoes with and without midgut breakthrough at 23 °C, 28 °C and 32 °C, we determined the optical concentration of DENV-2 to be 8.625 log_10_ TCID_50_/mL based on preliminary experimental results, with a time to harvest of 7 days post-infection. The MIR and MBR of *Ae. albopictus* were increased following a rise in temperature. These results showed that higher temperature facilitated viral replication for midgut breakthrough. The trends of the results were consistent with those of previous experimental research and model predictions [16, 25, 26].

Previous research demonstrated that temperature affects *Ae. aegypti* gene expression after Zika virus infection [19]. In the present study, the expression profiles of the *Ae. albopictus* midgut clustered after the mosquito ingested a blood meal containing DENV-2. Furthermore, temperature affected midgut transcriptome clustering in *Ae. albopictus* of the MB and MNB groups, as low temperature resulted in more DEGs in these. To better understand the relationship between temperature and DENV-2 infection in *Ae. albopictus*, we used WGCNA, which has been proven to be an effective method for assessing gene coexpression networks and hub genes in tumors, plants and parasites, etc. [27–31]. In this study, the highest correlation with temperature was observed for the ME3 module.

The mosquito transcriptome changed in response to DENV, which might be related to the mosquito’s antiviral system. In contrast to the innate and adaptive immunity of humans to resist the invasion of pathogens, mosquitoes lack adaptive immunity and mainly rely on innate immunity to suppress viral proliferation. RNA interference, Toll, IMD, Janus kinase pathway signal transduction and activation and other pathways play important roles in mosquito antiviral immunity [32]. The gene expression profiles of mosquitoes infected with viruses were transformed following temperature change. In a previous study, *Ae. aegypti* infected with chikungunya virus were cultured at 18 °C, 28 °C and 32 °C; the Toll, IMD and Janus kinase pathway signal transduction and activation pathways were upregulated at 28 °C, and high temperature appeared to damage the mosquito’s immune defenses [33]. In our study, the pathways of the ME3 module regulated by temperature included RNA degradation, the Toll pathway and the IMD pathway.

DENV is an RNA virus, and the RNA degradation pathway is closely related to the proliferation of DENV in mosquitos [34]. Among the genes we identified, it has been shown that heat shock cognate 70 protein interacts with chikungunya virus to promote its entry into C6/36 cells [35], and 70-kDa heat shock cognate proteins have been identified as the most critical components in DENV-4 binding and entry into C6/36 cells [36]. Additionally, CCR4-NOT transcription complex subunit 6-like belongs to the CCR4-NOT complex family, which is involved in transcription, translation and messenger RNA decay [37]. The expression levels of CCR4-NOT complex genes were upregulated in DENV-infected cells, which is conducive to the proliferation of DENV [38]. In this study, the CCR4-NOT complex genes were highly expressed at 32 °C, which suggests that DENV-2 proliferated more quickly at 32 °C, which helped DENV-2 break through the midgut barrier.

Toll-like receptors are pattern recognition receptors that recognize mosquito-borne viruses and promote the maturation of Spätzle. The interaction between Toll-like receptors and Spätzle involves myeloid differentiation primary response protein 88 (MyD88), followed by the activation of the transcription factor nuclear factor-kappa B, which induces the release of nuclear antimicrobial peptides and other antiviral molecules [39]. Lower DENV-2 loads in mosquitoes from southern and western China may be related to the innate immunity of mosquitoes as affected by the Toll pathway [40]. Three proteins are regulated by temperature: modular serine protease, peptidoglycan-recognition protein and MyD88. MyD88 serves as the key mediator of Toll signaling. The inhibition of MyD88 significantly enhances the replication of DENV-2 in *Ae. aegypti* [41]. MyD88 is also involved in the antiviral immunity of *Ae. aegypti* against Japanese encephalitis virus [42]. We speculate that the MyD88 molecule is key to the virus’s ability to break through the midgut barrier.

Functional validation of the temperature regulation of the genes coding for these three proteins was not undertaken in the present study. Thus, in future work, we will produce the small (short) interfering RNA and double-stranded RNA of these genes to detect the proliferative ability of DENV-2 at the cell level and analyze the vector competence of *Ae. albopictus* to transmit DENV-2 at different temperatures.

## Conclusions

To explore the mechanism through which temperature affects the transmission of DENV-2 by *Ae. albopictus*, we examined the variations in transcriptomes of its midgut at different temperatures using RNA sequencing. The altered genes identified here may be involved in viral resistance or viral infection, and *Ae. albopictus* may be more susceptible to DENV-2 when these genes are altered. Some important pathways, including the RNA degradation, Toll and IMD pathways, were identified by WGCNA and KEGG analysis as being regulated by temperature. This study provides experimental evidence applicable to the prevention and control of dengue.

## Supplementary Information

**Additional file 1: Figure S1.** The Pearson correlation coefficient of each sample was analyzed by heatmap.

**Additional file 2: Figure S2.** The ME3 module correlating with temperature was evaluated by GO analyses.

**Additional file 3: Table S1.** Primers used for qRT-PCR.

## Data Availability

Publicly available datasets were analyzed in this study. Sequencing data were deposited in the NCBI Sequence Read Archive under accession no. PRJNA772927.

## Abbreviations

CT: Control
DEG: Differentially expressed gene
DENV: Dengue virus
FPKM: Fragments per kilobase of transcript per million fragments mapped
GO: Gene ontology
IMD: Immunodeficiency factor
KEGG: Kyoto Encyclopedia of Genes and Genomes
MB: Midgut breakthrough
MBR: Midgut breakthrough rate
MIR: Midgut infection rate
MNB: Midgut nonbreakthrough
MNBR: Midgut nonbreakthrough rate
MyD88: Myeloid differentiation primary response protein 88
PCA: Principal component analysis
PCR: Polymerase chain reaction
qRT-PCR: Quantitative real-time PCR
TCID_50_: 50% Tissue culture infective dose
WGCNA: Weighted gene correlation network analysis
