# Supplementary figures and images for: Transcriptome analysis of Aedes albopictus midguts infected by dengue virus identifies a gene network module highly associated with temperature
Source: Parasit Vectors. 2022 May 19;15:173. doi: 10.1186/s13071-022-05282-y (PMC9118615; doi:10.1186/s13071-022-05282-y)

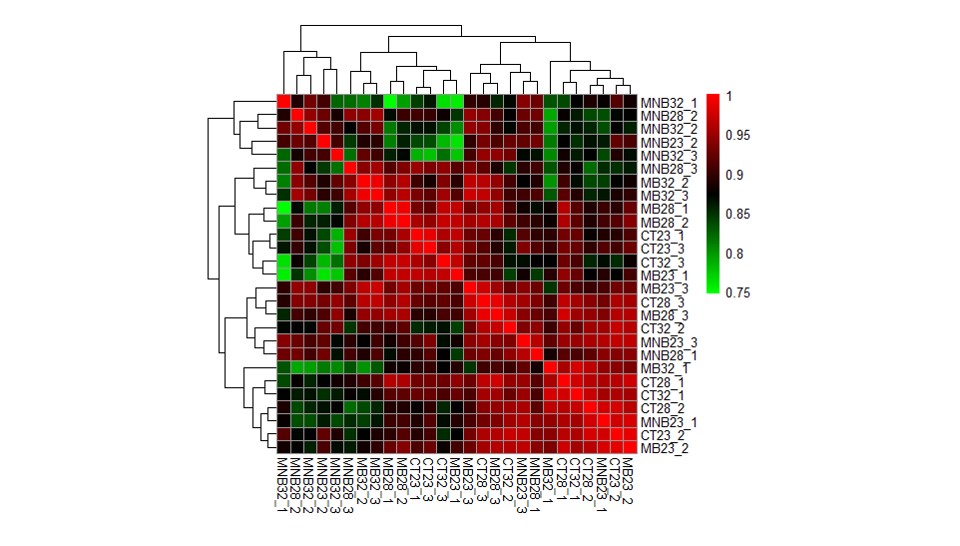

Supplement: Supplementary file 1 — Additional file 1: Figure S1. The Pearson correlation coefficient of each sample was analyzed by heatmap. [file 13071_2022_5282_MOESM1_ESM.jpg]

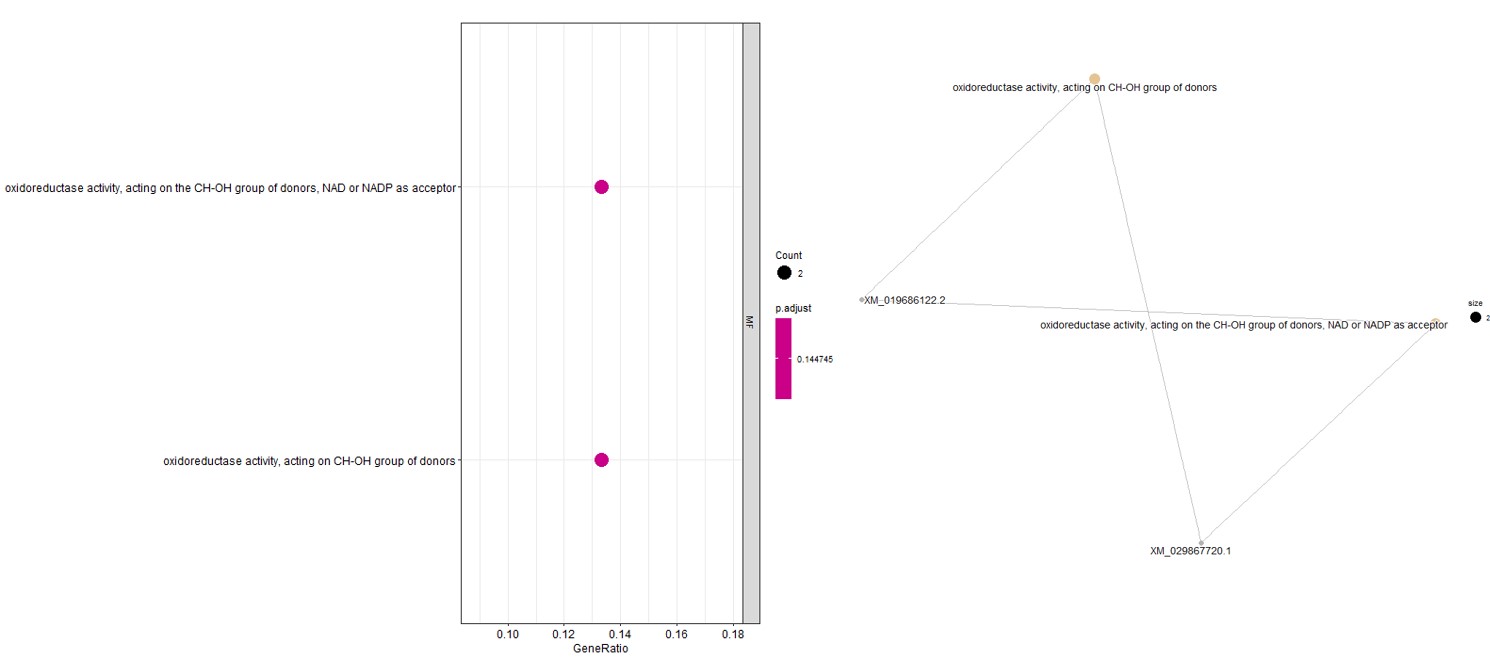

Supplement: Supplementary file 2 — Additional file 2: Figure S2. The ME3 module correlating with temperature was evaluated by GO analyses. [file 13071_2022_5282_MOESM2_ESM.jpg]
